# Treatise on the Diseases of the Kidneys and the Morbid States of the Urinary Secretion. With Folio Plates

**Published:** 1843-04

**Authors:** 


### ART. XV.

*Traité des Maladies des Reins, et des Altérations de la Sécrétion Urinaire, &c. Avec un Atlas in Folio.* Par P. RAYER.—*Paris*, 1841. Tome III. pp. 810.

*Treatise on the Diseases of the Kidneys and the Morbid States of the Urinary Secretion. With Folio Plates.* By P. RAYER, Physician to the Hospital la Charité, &c.—*Paris*, 1841. Vol. III.

M. RAYER grows upon our esteem. Each new volume of his admirable work, instead of exhibiting marks of its author's weariness, proclaims the vigour of his untiring industry; instead of occasionally giving hint that his curiosity in clinical research begins to flag, uniformly bears testimony that appetite for inquiry may grow by what it feeds on. And when we reflect that a work of such magnitude has been steadily continued, in spite of the constant occupation of time by extensive hospital and private practice, we are tempted to regard its author with stronger admiration than, perhaps, in sober reason, can ever be believed the meed of literary labour.

PYELITIS. Having in his former volumes disposed of the inflammations of the proper tissues of the kidneys themselves, M. Rayer proceeds in that now before us to examine the inflammatory affections of the structures more or less intimately connected with those organs. Inflammation of the pelvis and calices first engages his attention; and of this disease the author may be said (if it be allowable for a moment to adopt the usual but rather pompous phraseology of our neighbours under such circumstances) to have created the history. Pyelitis, the name proposed for the affection, (from πυελος, pelvis,) is in truth, in all that regards precise knowledge of its seat and anatomical characters, but more especially of its symptoms, essentially a novelty to the observer of renal disease. It was invariably confounded during life with actual nephritis, and is still so confounded by the majority of persons; while on the other hand it is not a little amusing to find certain writers, after having had their confusion of ideas on the subject cleared up by the lucid exposition of M. Rayer, suddenly discovering that this very pyelitis was among their oldest pathological acquaintances. That these writers, whose conscientiousness we by no means desire to gainsay, had seen cases of pyelitis before M. Rayer wrote is indubitable; but that they had acquired the power of distinguishing it from nephritis, that they even suspected the existence of such a distinct and in many respects totally different affection, is what, until proof of the affirmative be produced, we conceive ourselves at liberty to doubt altogether.

*Pathology and diagnosis.* Pyelitis, occasionally occurring as an acute, is most commonly a chronic, disease; and may be considered to be of various species according as the character of the inflammation and the nature of the causes producing it vary; thus we have the simple, calculous, blenorrhagic, gangrenous species, &c. The disease may implicate the entire of both pelves, or a part only of one; it may even be confined to one or more calices.

The anatomical characters of the *acute* form of the affection may be stated as follows: General or partial injection of the mucous membrane, accompanied occasionally with minute petechiæ, or effusions of blood sufficiently considerable to deserve the name of ecchymoses, and in more rare instances with accumulation of a certain quantity of blood in the cavity of the pelvis or calices, are among the more remarkable appearances. In some cases partial deposits of whitish or grayish pseudo-membranous matter are found adhering more or less closely to the surface, a circumstance of importance, as contributing to show that the tendency to formation of plastic exudation, though rarely brought into action on mucous surfaces, yet exists in them all. When the accumulation of this matter is sufficiently abundant to impress a special character on the inflammation, when this is in fact *pseudo-membranous*, the orifices either of the calices, pelvis, or ureter may be blocked up and effectually obstructed; this is the only circumstance under which such obstruction occurs in the acute disease, as thickening of the mucous membrane itself is generally of slight amount. When caused by retention of urine, acute pyelitis may be attended with dilatation of the pelvis and calices and flattening of the mammillæ. The urine in those cases is invariably mingled with pus or muco-pus, discoverable with the microscope when otherwise invisible. The other physical and chemical properties of the fluid vary with the species of pyelitis; it may contain urates, uric acid, phosphates, albumen, &c.

In *chronic* pyelitis, the most common colour of the mucous membrane is dull white; varied here and there with reddish or more frequently brownish injection; if the disease be of very old standing, grayish or slate-coloured patches are far from uncommon appearances. The veins on the exterior of the kidney are sometimes much enlarged, and so arranged as to form net-works. The mucous membrane may have undergone thickening to such an amount as to entail contraction of the orifices of the calices, and even to cause an apparent conversion of those canals into fibrous tissue. We strongly doubt this conversion as described by M. Rayer, and apprehend that in such cases the mucous membrane has been originally destroyed by ulceration or otherwise, and exudation or plastic matter, thrown out on the new surface, hardened into the fibrous-like tissue he has figured. This is probably at least the most common case; there are others no doubt in which the exudation of similar matter *under* the mucous membrane leads to a like result as regards apparent thickening of the mucous membrane and obstruction of the channels for the passage of the urine.

A curious condition observed by M. Rayer in both forms of pyelitis, but in most rare cases, is an eruption of transparent vesicles like the sudamina so common in the typhoid fever of Paris.

Ulcerations of various extent and depth add to the amount of organic change in many cases. They vary in size from a pin's head to that of a sixpence, or more, and may spread so deeply as to cause complete perforation of the pelvis and the establishment of fistula, communicating with the surrounding cellular membrane, the peritoneum, the duodenum, the large intestine, &c. It is important to remember that these perforations may occur quite independently of distension of the pelvis, although renal fistulæ do certainly occur most frequently as the final morbid change in cases of atrophy of the kidney, with distension of the pelvis into a huge multilocular cavity. These ulcerations, more especially when of moderate dimensions, are susceptible of cicatrization, and when so cured present precisely the anatomical varieties observed in healed ulcers of the stomach, and, we may add, of mucous membranes generally.

With the enormous tumours produced by distension of the pelvis with urine, pus, blood, &c., most persons are tolerably familiar, at least as regards the fact that tumours of this description, extending from Poupart's ligament below, to the upper limits of the abdomen above, and driving the liver or spleen upwards into the chest, are not very uncommon morbid appearances. The atrophy of the renal substances, which coadvances with the accumulation in the pelvis, until this is distended into a simple membranous sac, readily explains the error of the older observers, ascribing these changes all to ulceration and suppurative destruction of the kidney itself. There is an infinitely rarer species of atrophy of this organ, pointed out by M. Rayer, in which it retains its natural form, while gradually reduced to a less size than that of the kidney of a new-born infant, the pelvic accumulation proceeding as in the ordinary cases.

Chronic pyelitis may be of cancerous or tuberculous origin. In the latter case M. Rayer, as Dr. Carswell had done previously, finds "tuberculous matter" adherent to the mucous surface. We shall not stop here, however strongly tempted, to argue the question of the absolutely tuberculous nature of this matter; for we could not give fair expression to our convictions on the subject, without entering into a disquisition upon the meaning of the word tubercle (or rather upon the nature of the matter of tubercle), in nowise compatible with the general subject now under consideration. We must venture, however, to question the justness of MM. Carswell and Rayer in terming this substance tuberculous, if by the word we are to understand matter of the same essential nature as the well-known pulmonary product.

Practically considered, there is a very natural division of all cases of pyelitis into those produced by foreign bodies, and those occurring independently of any such influence, as far as our senses are capable of deciding. And of all foreign materials generating the disease, calculi are beyond comparison the most frequent and the most important. We shall not enter upon a description of renal gravel and calculi, because these products are generally known in their essential characters, but in tracing, with M. Rayer for our guide, the history of pyelitic symptoms thus originated, refer briefly to the different physical and chemical forms under which calculous deposits occur in the kidneys.

1. When gravel or very minute calculi pass from the calices into the pelvis, thence without great difficulty into the ureter, and eventually reach the bladder, severe pain is felt, but it exists independently of inflammation of the mucous surface. But if a calculus be too large to force its way from the calices to the bladder, it gives rise to symptoms referrible to four distinct types, according to their severity and duration.

2. The least severe type is that commonly known as nephritic colic; the urine is diminished in quantity, and invariably contains mucus and blood; the latter may be very small in quantity, but the characteristic globules may be readily detected with the microscope. These conditions of the urine, together with the better known pain passing from the kidney to the bladder, vomiting, fainting, &c., cease with the arrival of the calculus in the bladder.

If, on the contrary, the calculous heap or masses, unable to make their way from the renal cavities, remain there, chronic pyelitis is one of the inevitable results. Pain of a dull kind, in the loins, increased by movement and by various modes of pressure acting directly and immediately on the part, or from a distance, diminished in the recumbent posture, extending along the ureter to the bladder and testicle, is one of the most constant symptoms. This pain is subject to occasional exacerbation, when the urine becomes reddish, bloody, and coagulable by heat. The condition of the urine varies in different *emissions;* sometimes bloody, or containing notable quantities of mucus, at other times perfectly transparent. It is acid, if the gravelly or calculous matter is composed of lithic acid; alkaline, if this consist of phosphates.

3. In the third state irregular rigors, of frequent occurrence, are observed; the urine, sometimes bloody, is more commonly whitish and turbid, and deposits a greenish white sediment, composed of pus and salts. All this may occur, without the formation of appreciable tumour, and the case terminate fatally by exacerbation, attended with increase of pain, diminished secretion or suppression of urine, nausea, vomiting, fever, &c.

4. Another condition of the disease is marked by the coexistence of purulent urine and renal tumour,—a tumour which may acquire such magnitude as to weigh from ten to fifty pounds. Posteriorly this tumour gives a dull sound under percussion, as likewise anteriorly, unless the colon in front of it be distended with gas: on the right side it may eventually push away the colon, and form a continuous mass with the liver; to mark out its limits becomes, under these circumstances, impossible. When the mass has attained a certain bulk, it always appears tuberculated, and is the seat of obvious fluctuation. Pain appears rarely to exist with any severity; some patients fancy they feel calculi moving about in the tumour, and a particular sort of shock or fremitus has, it is alleged, been felt by observers. M. Rayer doubts the accuracy of the latter statement, and appears to believe its authors deceived by friction of the anterior wall of the abdomen against the surface of the tumour.

It is a matter of great practical importance to bear in mind that the physical properties of the urine are liable in this affection to vary greatly within the same twenty-four hours: it may be at one time loaded with pus, at another present its natural aspect. M. Rayer appeals to the easy explanation of the peculiarity afforded by supposing the urine to be at one time furnished by the healthy, at another by the diseased kidney. This explanation assumes that such variation in the condition of the urine does not occur where both kidneys (as is rarely the case, however,) are implicated.

The coagulability of the urine by heat and nitric acid is not in the direct ratio of the amount of pus it contains.

5. The fifth condition described by M. Rayer is that of atrophy of the kidney, without purulent secretion. This state occurs where the mucous membrane of the calices and pelvis in contact with a large-sized calculus becomes hardened, thickened, and so altered in structure as to lose its power of secreting. If the opposite kidney be healthy, there are no known means of detecting this morbid state during life; and cases are not exceedingly uncommon in which an amount of secretion, little if at all below the natural standard, has been maintained by means of an hypertrophous condition of the other organ.

The diagnosis of calculous pyelitis is most minutely investigated by M. Rayer, each of its symptoms being separately considered in its points of resemblance and dissimilarity to the same symptom when produced by other affections. Lumbar pain, a prominent symptom of pyelitis, may exist in nephritis of various kinds, in cases of hydatids of the kidney and ureter, in nephritic colic, in retention of urine without inflammation, in lumbago, in certain diseases of the lumbar spine, in psoitis, in aneurism of the descending aorta, in pregnancy, in certain diseases of the uterus, in certain cases of partial peritonitis, and in inflammation of the subperitoneal cellular tissue of this region. We cannot follow M. Rayer in his enumeration of the distinctive characters of pain originating from each of these causes, but select the paragraph referring to lumbago as an example of the whole:

"In lumbago the pain commonly affects both sides at once, and with about equal intensity; it is always increased by movement of the trunk; most generally it is unattended with fever, and sometimes preceded by other muscular or articular pains. In pyelitis the pain is almost always felt on one side only (double calculous pyelitis is rare), or at least with unequal intensities on both sides; it is increased, no doubt, by muscular contraction, but not with anything like the severity felt in the instance of lumbago. However, it is not always easy to distinguish the two species of pain." (p. 31.)

That renal calculi, of considerable size, may exist without causing pain, is demonstrated by not a few cases reported, with the results of dissection, by Baglivi and others.

The excretion of purulent urine is not peculiar to pyelitis. But the affections which may be attended with this symptom are some of them so rare—mere accidents in pathology, indeed,—that it is unnecessary to examine particularly into their characters; to this class belong abscess of the subperitoneal cellular tissue opening into the urinary passages, suppurative psoitis, or accumulations of pus in the ovary, following a similar course. Cystitis, on the other hand, whether acute or chronic, is a comparatively common affection, and leads likewise to impregnation of the urine with pus. When, in addition to absence of pain and tumour in the loins, the urine is stringy and viscid, the affection is commonly set down as vesical; but it must be remembered that this viscidity is the mere result of the action of alkali on pus, and would occur in the case of pus conveyed from the calices and pelvis, provided the urine were alkalescent. Besides, pus discharged from the bladder is not always ropy. These facts, insisted on by M. Rayer, show the importance of not trusting to the mere condition of the excreted pus as a distinctive sign of the two maladies, when information can be derived from other sources; nevertheless we are satisfied from the author's own descriptions, and our own comparatively limited experience, that the conditions referred to are extremely valuable guides in the distinction sought to be established. M. Rayer raises his voice against the opinion that when derived from the kidney the purulent matter is voided after the urine, or at the end of micturition; he maintains, and this we have likewise seen, that whatever be its source, it is voided mixed with the urine, and discharged only more abundantly at the close of the act.

Our author next enters into a most elaborate examination of the distinctive signs of all tumours which may be confounded with that produced by pyelitis, with retention of pus, urine, blood, &c. in the distended pelvis. We cannot follow him through the abundant details furnished under this head, but shall extract the list of affections capable of giving rise to error of diagnosis, and then add a specimen of the manner of distinguishing them:

"The tumours capable of being confounded with those produced by chronic calculous pyelitis are, on the left side, all those resulting from morbid development of the spleen; on the right side tumours of the liver and of the gall-bladder; on both sides various renal tumours of a different nature (hydronephrosis, abundant accumulation of blood in the calices and pelvis, cancer and tuberculous disease of the kidney, acephalocysts in that organ, &c.,) extra-renal abscess either idiopathic or consecutive to perforation of the kidney, of the colon or cæcum; abscess from caries of the spine; tumours proper to the supra-renal capsules; aneurism of the aorta; encysted tumours containing various fluids or acephalocysts; and lastly stercoral tumours produced by the accumulation of fæces in the colon and cæcum." (p. 37.)

The distinction of the last-named tumour from a renal tumour produced by chronic pyelitis is not so easy always as might on first consideration appear, especially if fecal accumulation have occurred in a person who had previously laboured under urinary symptoms.

"However, tumours formed by the ascending or descending colon obstructed with feces, are commonly more elongated, less broad, and of a more distinctly cylindrical form than renal tumours. On the right side stercoral tumours are commonly prolonged towards the cæcum, which gives a dull sound in some points, a clear one in others from distension with gas. On the left side the stercoral tumour passes towards the iliac fossa and cavity of the pelvis. We may often ascertain at the same time that the transverse colon contains hardened fæces. Stercoral tumours are more common on the right than the left side, and when they are painful to the touch and under pressure, this is generally more marked before than behind; while the contrary is, in the majority of cases, the fact in morbid dilatations of the kidney, when there is no inflammation of the corresponding part of the peritoneum. Lastly, stercoral tumours disappear after one or two active purgations." (p. 44.)

*Treatment.* The directions given by M. Rayer for the treatment of pyelitis in the different states already referred to, are sound and judicious; but as they present little that is actually novel or otherwise very interesting, with the exception of his remarks on the subject of nephrotomy, we shall limit to these our notice of this portion of his chapter.

*Nephrotomy.* If a pyelitic collection of the kind we have been considering exists in a subject otherwise of a good constitution, and is habitually painful and the source of fever and of derangement of the stomach and bowels; if the pain frequently increase in violence and is attended with complete suppression of urine, or symptoms of inflammation of the surrounding parts, the operation of nephrotomy, notwithstanding its difficulty and the chances of failure, should, according to M. Rayer, be practised. The indication for the operation will of course be stronger if there be extra-renal abscess or perforation of the sac formed by the distended pelvis. The author's motives for giving this advice are that, left to themselves, pyelitic collections are almost invariably fatal, unless they open externally by spontaneous perforation; that the operation itself presents no immediate danger; no large vessels are exposed to the knife, there is no abundant hemorrhage, at least in the great majority of cases, to be feared, and no risk of opening either the peritoneum or intestine incurred. We must refer to the original work for most precise directions as to the method of fixing the situation for performing the incision, &c. The only real objection to the operation M. Rayer considers to be the difficulty of breaking down and extracting such calculi as may be contained in the pelvis, and this part of the operation may be deferred, as the immediate and essential point is gained, the evacuation of the fluid collection in the cavities of the kidney. M. Rayer refers to numerous cases of indubitable success on record, as forming the final justification of nephrotomy under the circumstances; but, as he himself admits, in some of these instances the operation performed did not in reality deserve this name, as the calculus had either already escaped spontaneously into an extra-renal abscess or the surgeon had contented himself with incising the walls of an extra-renal abscess, leaving the perforation of the pyelitic sac and the escape of the renal calculus to be accomplished by a natural process.

Such being the circumstances which, according to the learned author, warrant the operation, the absolute contraindications claim to be mentioned. These are the existence of renal calculi upon both sides, (unless, however, in the case of *extra-renal* abscess, which should always be opened;) the circumstance of the pus flowing freely from the pelvis into the ureter, without the existence of renal tumour or fair motive for apprehending rupture of the kidney, and above all of a good state of the general health justifying the notion that the unaffected kidney supplies by increased action the want of the diseased one; and finally the fact of the existence of severe organic disease either in other parts of the urinary organs or in other viscera.

Of the three methods employed for effecting an opening into the distended pelvis, namely incision, incision of the external parts and puncture of the sac with a trocar, deep cauterization followed by incision, M. Rayer prefers the former. For considerations regarding the mode of operating, the extraction of the calculus, &c., we refer to M. Rayer's own pages.

M. Rayer next proceeds to examine the peculiarities of other species of pyelitis; to these as described in the original we recommend particular attention, as they are more especially the varieties of the affection, (e. g. simple, verminous, &c.,) to which our opening remarks respecting their novelty to practitioners refer. A series of admirable cases and an historical sketch of the progress of knowledge respecting calculous pyelitis concludes this division of the subject.

PYELO-NEPHRITIS. Under this name M. Rayer designates the association of inflammation of the renal tissues and of the pelvis and calices, which is of more common occurrence than either of these affections separately. The pelvis and calices are generally first seized; the renal substances rarely earliest implicated. The causes of the disease are those of simple pyelitis; it may run an acute or chronic course, and may be simple, calculous, albuminous, or hemorrhagic and gangrenous. In these cases there is a combination of the purulent urine of pure pyelitis with the other morbid states of that fluid and the *general symptoms* observed in nephritis; these symptoms do not exist in pure pyelitis. In the treatment of the double disease it is in the great majority of cases important to attack the inflammation of the renal substances first.

PERINEPHRITIS. This is the name given by M. Rayer to inflammation of various kinds of the tissues—adipose, cellular, and fibrous—surrounding the kidneys. This affection may be secondary, succeeding then to nephritis or caused by infiltration of urine; or it may be primary, produced by contusions in the loins, the impression of cold or damp, &c. When *acute* and recent, the delicate cellular tissue interposed between the kidney and its fibrous capsule is traversed by vessels abundantly injected, sometimes infiltrated with serosity or pus, or the latter is collected into small abscesses. The accumulation of pus may, observes M. Rayer, be so considerable as to cause rupture, by distension, of the capsule. The external cellular tissue, especially that behind the kidney, sometimes becomes the seat of collections of pus; the lumbar subcutaneous cellular tissue is infiltrated with serosity, and this œdema combined with fluctuation furnishes one of the best signs of extra-renal abscess. Accumulations of this kind may eventually communicate with the intestine or the bronchi.

In cases of *chronic* perinephritis the subfibrous cellular tissue of the kidney may grow remarkably thick, either generally or in various points, which may have a black colour and form prominences on the surface. The fibrous capsule may adhere so firmly that it cannot be removed without tearing; it may be the seat of cartilaginous and osseous deposits.

*Diagnosis.* There may be very great difficulty in the diagnosis of *primary* abscess of the cellular tissue around the kidneys. The pain complained of is more deeply seated than in lumbago; the lumbar region is not altered in shape; if there be fever the urine is red, as in inflammatory diseases, without presenting any of the characters peculiar to affections of the kidney or pelvis; the pain spreads; the fever increases; subsequently the flank becomes arched and œdematous, and percussion (which is painful) shows that the enlargement has been chiefly affected posteriorly. Collections of this kind should be opened the moment their nature is ascertained; they generally do well, in surgical phrase, and cicatrize with satisfactory speed. When the abscess is consecutive to calculous pyelitis, on the contrary, fistulæ often delay the progress of the case to cure for years.

RENAL FISTULA. We next come with the author (passing over his collection of cases of perinephritic abscess) to the history of renal fistulæ. These fistulæ are generally induced by the presence of one or more calculi in the pelvis or ureter, and may open either into the extra-peritoneal cellular tissue, or externally in the lumbar region or the crural arch, or into the colon, the duodenum, the cavity of the peritoneum, or finally into the pleura or lung of the corresponding side. Examples of each of these species of fistulæ are related by M. Rayer, derived either from his own experience or from periodical records. We recommend to the curious in pathological anatomy, a careful perusal of the cases of reno-pulmonary fistula, which we regret our limited space forbids us to extract.

RENAL HEMORRHAGE. The important subject of renal hemorrhage receives full investigation at the hands of M. Rayer in a chapter exhibiting his excellent powers of precise description and methodic arrangement to the very best advantage. Renal hemorrhage is of three distinct kinds: it may be symptomatic of certain lesions of the kidney; it may be symptomatic of certain general diseases; and finally it may be idiopathic.

The anatomical characters of renal hemorrhage are minutely described: we must content ourselves with a reference to all varieties except that termed *renal apoplexy*, which we have not seen elsewhere distinctly mentioned.

"On the surface of the affected kidney, knotty, irregular tuberculated eminences are perceived, some of them of deep black colour, others of chamois-coloured tint, more or less pure or variegated with black parts; all, or almost all, these eminences are surrounded by deep brown lines. Under a lens the substance of the kidney appears swollen with black blood; its tissue is granulated; neither clots nor lacunæ resulting from the absorption of the liquid can be anywhere perceived; the blood is infiltrated and ultimately combined with the renal substance."

The last sentence seems to involve an error, the more requiring correction because a common one: it is not possible in truth that blood, or any other material, should be at once infiltrated through and combined with a tissue.

Bloody urine coagulates with heat and nitric acid, and presents under the microscope the characteristic blood-disc, which acquires an irregular contour after some sojourn in the urine. Pure unaltered blood, or nearly so, is very rarely voided, unless in cases of wound or laceration of the urethra: in abundant renal hemorrhage the blood commonly coagulates in its passage, either in the ureters or bladder. In some cases the urine contains so small a quantity of fibrine and discs that it has a very slight pink colour only, and deposits no coagula, and it would be impossible to affirm that such urine contains blood without the aid of the microscope. Of the importance of this observation we are fully persuaded, having ourselves detected blood-globules in some quantity in the urine of a patient who, for several weeks continuously, passed urine which presented *no* appearance of impregnation with blood to the naked eye. M. Rayer well observes too that the daily quantity of blood voided in a case of renal hemorrhage cannot be determined by the inspection of one or two specimens of the urine. This fluid may sometimes be discharged transparent; when a huge clot fills the bladder almost, and when a coagulum accidentally blocks up the ureter on the side of the kidney furnishing the hemorrhage, the other organ of course furnishes natural urine, which might betray the physician into the notion that all hemorrhage had ceased. It must, however, be admitted with M. Rayer, that the variations observed in the quantity of blood passed at different periods during the day in renal hemorrhage are not always explicable; he has found nevertheless that it was more considerable than usual in the urine voided about three hours after meals.

The duration of renal hemorrhage is with difficulty foreseen. Hematuria sometimes ceases in a day or two in cases of calculous pyelitis; in other instances continues for months together. In cancers of the kidney, hemorrhage may be a persistent symptom, varying no doubt much in its amount from day to day, or week to week, but never wholly ceasing. The extent of the hemorrhage may be so great as to prove fatal; such is the immediate cause of death in some cases of cancer of the kidney; and idiopathic renal hemorrhage sometimes has proved similarly destructive to life.

A question of very considerable practical importance is,—being given a case of hematuria, how is the source of the hemorrhage to be determined? The circumstances leading to the belief that the blood comes from the kidneys are the existence of the feeling of a weight or dull pain, increased by firm pressure, in one or both renal regions; the absence of all organic lesion of the excretory organs of the urine; and in other cases the existence of some general malady of the system which experience has shown to be not uncommonly attended with effusion of blood from the kidneys. It must be remembered, however, with regard to the first of these characters that in "essential" renal hemorrhage the renal regions may be perfectly indolent, even under the strongest pressure.

Hemorrhage from tne ureters is rare; the only cases in which M. Rayer has observed it were examples of calculous ureteritis; and in two cases of the kind where the hemorrhage was frequent and abundant, the blood was furnished in great measure by fungous vegetations seated in the neighbourhood of a calculus. As during life both the ureter and the kidney were the seat of pain, the former from the calculus and fungus, the latter from the retention of urine, it was impossible to determine by which the blood was furnished.

With the exception of cancerous fungus of the bladder, and calculous and tuberculous cystitis, there are few affections of that viscus which give rise to hematuria; and the diagnosis of these is rarely, according to M. Rayer, attended with difficulty. We confess that this alleged facility is more than we could, either from our own capabilities or from what we have recognized to be the share of other persons, have anticipated to exist in respect of tuberculous cystitis. The detection of the other maladies is in truth easy to all.

When the blood comes from the urethra it flows without the patient's passing urine; and urine removed from the bladder by catheterism contains no blood, unless the lesion of the urethra be near the neck of the bladder, or the blood in consequence of some obstacle not being able to make its way outwards, regurgitates into the bladder.

In cases of renal hemorrhage the effused blood sometimes, either in consequence of its coagulation, or of some obstruction to its escape, accumulates in the pelvis and calices, and distends these cavities enormously. Walther published, in his book on Renal and Vesical Diseases, the very remarkable case of a young girl whose abdomen was sufficiently large to simulate pregnancy of seven months' duration (she was universally believed to be pregnant), and in whom the enlargement was, after death, found to depend upon enormous accumulation of blood in the kidneys; the hemorrhage was caused by calculi. Other cases, similar in the main, are on record.

Hemorrhages symptomatic of renal injury or disease are among the most important in their relations, if not from their actual amount, which fall under the notice of the physician. We need not return to the subject of injuries of the kidney causing hematuria, noticed in one of our former articles, except for the simple purpose of reminding the reader that nephritis (traumatic) is a very common complication in such cases, and requires the practitioner's attention after the bleeding has been arrested.

All species of nephritis may be accompanied with renal hemorrhage. Even the *simple* inflammation is much more commonly so attended than is usually imagined; and it is important to observe that in albuminous nephritis (Bright's disease) the urine frequently contains blood-discs, when not obviously discoloured to the naked eye. In rheumatic and gouty nephritis, hemorrhage similarly attends the acute period of the disease, and passive hemorrhage occurs in nephritis produced by the action of "morbid poisons" on the system.

Calculous pyelitis is one of the most frequent sources of this hemorrhage, as might be deduced from much that has been already said; and the verminous species (produced by strongyli—a rare entozoon, however, in the human subject), has been not unfrequently known to give rise to hemorrhage.

Of degenerations of the kidney causing effusion of blood, the cancerous and tuberculous are by far the most important. In another part of this article we shall have occasion to return to the subject.

Of the general affections of the system capable of causing effusion of blood from the kidneys, some are of the hemorrhagic species,—for example, purpura and scurvy. Hematuria is much more common in the former of these diseases than the latter; and it is curious to observe the manner in which, as the hemorrhage ceases, the different elements of the blood disappear from the urine,—first, the fibrine ceases to be discoverable, then the blood-discs, while the albumen remains still sometimes readily discoverable. Such is, no doubt, the ordinary course of things in hematuria of every kind originating in the kidneys.

The tendency of numerous substances injected into the veins to pass off by the kidneys is well known, and it is very remarkable that in animals submitted to transfusion, the blood transudes through the kidneys in numerous cases. Of fifty animals thus experimented on, twenty, according to P. Frank, had hematuria.

The eruptive fevers, yellow fever, and continued fever, are not unfrequently accompanied with bleeding from the kidney; and there are certain agencies of less importance, which, after having acted upon the system generally, and on the blood in particular, determine hematuria, commonly of slight amount. Thus, according to M. Rayer, individuals struck with lightning have been known to pass blood with the urine; and the effect of certain substances, as cantharides, in this way, is well known;—the use of balsam of Peru, garlic, onions, asparagus even, in large quantities, is said to be followed by a similar result. M. Rayer has met with one case of lead-colic attended with hematuria, and that the latter depended upon the same cause as the former—the absorption of lead—seems proved by the fact, that with the decrease of the colic the discharge of blood gradually diminished also. It is much more common, according to this author, to detect albumen than blood in the urine of these patients; but as in several cases observed by him the albuminuria persisted after the cure of the colic, it is more than possible that some amount of Bright's disease may have existed in the kidneys.

Idiopathic, or essential renal hemorrhage, that occurring independently of local, organic, or general disease, an affection of much rarity in these countries, but apparently common in certain tropical regions, is treated of under five different heads by our author. This species of hemorrhage may, he considers, be *continuous*, *intermittent* or *periodical*, *supplementary*, *critical*, and *endemic*.

1. As examples of the continuous essential variety two cases are related, in one of which a very slight degree of bleeding was remarked, while in the other the renal hemorrhage actually proved fatal. Whether the title of essential can be fairly given to the hematuria in these cases, seems matter of doubt. In the case of slight bleeding there was pain in one renal region, and the hemorrhage was stopped by antiphlogistic treatment; in the case of severe hemorrhage, there were manifest evidences of chronic renal inflammation detected after death. Nevertheless it may be urged, that in the last case the bleeding was altogether out of proportion to the inflammation, and could therefore scarcely be considered a mere dependence upon it.

2, 3. Of idiopathic *periodical* hemorrhage from the kidney, M. Rayer has himself observed no example, and believes that such a discharge, unless when *supplementary* of the catamenia, or hemorrhoids, or connected with intermittent fever, must be extremely rare. The interest of the information collected from various sources, on both these varieties of hematuria, will reward its perusal.

4. Upon *critical* hemorrhage, M. Rayer has nothing new to offer, and his collected materials are meager and unsatisfactory: at both these circumstances we by no means wonder; such hemorrhages have doubtless been often, if not always, imaginary, that is in their *critical* character.

5. M. Rayer's chapter on the endemic hematuria of the Mauritius is replete with interest; and although the subject may not have immediate practical importance in the eyes of persons practising in Great Britain, we have the advantage of our colonial readers sufficiently at heart to indulge them with a brief summary of M. Rayer's elaborate investigations of this very curious malady. It occurs especially in children, and in three principal forms,—those of simple hematuria; hematuria with uric acid gravel; hematuria with chylous (albuminous and fatty) urine. The influence of heat of climate in its production would, at first, appear obvious from the localities in which it occurs, (it is observed also in Nubia and Upper Egypt,) and its absence in temperate countries, were it not for the fact of its absence in so many other climates equally hot. The quantity of blood voided varies extremely; it is sometimes so small that no coagula are formed, and micturition is accomplished without pain; some emissions in the course of the twenty-four hours may be altogether bloodless. In the most favorable cases it would even appear that several days may from time to time elapse, without any appearance of blood in the urine, when at length the discharge recurs, without appreciable cause; children affected with this comparatively benign form of the disease are able to pursue their studies, and retain the outward habitus of health. In cases of greater severity, coagulation of the blood in the bladder may give rise to retention of urine and its consequences, while repeated loss of the vital fluid renders the child pale and languishing. Nevertheless M. Rayer is inclined to believe, both from what he has himself observed in natives of the island removed to France, and from what he has been able to ascertain, that loss of blood is rarely carried to such an excess as to render the constitution cachectic, and lead to general dropsy.

The sediment of the urine in this disease is almost wholly composed of blood, or of blood-discs and crystallized lithic acid, the latter accumulated sometimes in sufficient quantity to form gravel, capable of causing nephritic colic, and obstructing the secretion of the urine. The frequency of this gravel in the youth of the Mauritius makes it a question of interest whether adults suffer in a higher proportion from stone than those in other countries; this M. Rayer's information does not enable him to say.

In another form of the disease the patient passes two distinct kinds of urine in the twenty-four hours. One of them is ordinary bloody urine; the other, which appears to be usually elaborated some hours after digestion, is of a pale red colour. The latter separates, by standing, into two parts, the lower of which is bloody, the upper opaline, and of a milky white colour, or completely opaque. Examined under the microscope, this white portion is found to contain, commonly, blood-discs—and fatty matter not presenting the globular form may be removed by ether and evaporation; in some cases, however, no blood-discs are to be found. It invariably contains albumen, and is not deficient in urea. Compared with a mixture of healthy urine and rose-coloured chyle, taken from the receptaculum chyli in a horse, the resemblance of the two liquids—the artificial chylous urine and the natural chylous urine—struck M. Rayer as being most forcible. In both these were globules, having the aspect of those of the blood; in both there was albumen, and a small quantity of fibrine; and lastly, both contained a considerable amount of fatty matter. Further, albumino-fatty urine resembles urine to which the organic elements of the chyle, minus its globules, had been added; and "this species of chylous urine," adds the author, "considered in its relations to the chyle, was evidently analogous to albuminous urine derived from the blood." In two cases, referred to by M. Rayer, it was ascertained that this species of hematuria, with chylous urine, coexisted with a peculiar alteration of the blood,—this fluid resembling in its constitution the chyle of the thoracic duct.

Persons voiding chylous urine often enjoy apparently good health; gout and lithic acid deposit in the urine are sometimes observed as coexisting states.

The endemic hematuria of the Mauritius, whether simple or complicated with lithic-acid gravel, or replaced by chylous urine, always affects a chronic course. The hemorrhage sometimes ceases for several days, or even weeks, then returns, and continues with remissions or intermissions of variable length, for years. In addition to the influence of climate, already alluded to, on the production of this disease, it appears, from its persistence in some cases for years after the removal of the patient to Europe, that it may become a constitutional state.

M. Quevenne analysed the urine of a female, aged seventy-eight, who had voided albumino-chylous urine from the age of twenty-five to seventy-five; at the latter age, the renal discharge became natural, and the patient believed herself cured. After about a month spent at sea, on her voyage from Bourbon to France, the chylous condition returned with greater intensity than ever. Examined with the microscope, the fluid was found to contain altered blood-discs, globules like those of pus or mucus, and epithelium scales. On analysis 100 parts furnished,

| | |
|---|---|
| Aromatic fatty oil - - - - | 1·90 |
| Albumen - - - - - | 0·70 |
| Extractive and saline matters, divisible into extractive matter soluble in alcohol and lactate of urea - - | 1·20 |
| Extractive part, insoluble in alcohol and salts consisting of chlorides, phosphates and sulphates, and uric acid - - | 1·10 |
| Water - - - - - | 95·10 |
| | 100·00 |

M. Rayer observes (and the observation is perfectly applicable to England,) that although it is common in Paris to meet with affections in which the urine contains albumen, with or without blood-discs, true renal hemorrhage (comprising all varieties, of course,) is rare there. P. Frank has made a similar remark in respect of Vienna and Pavia. Of 1913 patients treated by him during seven years at Vienna, one only laboured under hematuria; of 13,467 persons dying in the general hospital at Vienna, one only was carried off by hematuria; and lastly, of 4000 persons affected with severe and rare diseases, and treated at the Clinical Institute at Pavia, in the course of ten years, six cases, only, of spontaneous hematuria occurred.

HYPERTROPHY OF THE KIDNEYS. This disease, that is, pure and simple enlargement without appreciable change of structure, is one of the rarer affections of those organs. It may be general or partial, and affect both or only one kidney. In almost all cases of absence of one kidney, the existing one is notably larger and heavier than natural, and may even weigh as much as both kidneys together of a subject of the same age; the caliber of the renal artery is then double that usually observed. This sort of excess of volume of one kidney is also noticed in many cases, where the other is small and rudimentary; and also where this other has been rendered unfit to perform its functions by disease. In cases of partial disorganization of the kidneys, the parts not implicated may become hypertrophous, whence arise curious alterations of shape of the organs, the nature of which does not always on first sight appear obvious.

Hypertrophy of *both* kidneys is sometimes a congenital state, when it invariably coincides with unusually great development of the renal arteries. This condition is likewise an observed effect of certain morbid states of the kidney attended with excessive secretion of urine—saccharine diabetes, for instance. In this disease, the hypertrophy implicates the cortical substance especially; the caliber of the vessels generally is also manifestly increased, and the corpora Malpighiana are more apparent than natural. As excess of functional activity on the part of the kidney may become a cause of its hypertrophy, so its hypertrophy may, in turn, augment the secretive action of the organ.

Hypertrophy of the kidneys has been met with in the fœtus: Osiander has even related a case in which both were so enormously enlarged as to obstruct parturition in the mother.

ATROPHY OF THE KIDNEY. The converse state, atrophy, is liable to affect the kidney under a considerable variety of circumstances, and may be limited to one, or a part of one organ, or exist in both. Atrophy, as a congenital state, may be explicable by unnaturally small caliber of the renal artery; or there may be no particular condition of that vessel capable of accounting for it. When developed after birth, atrophy of the kidney is the result of pressure. If this unnatural pressure be set in action at an early period of life, a suspension of development is the mode in which the atrophy is directly produced; if at a more advanced period, absorption of the renal tissue occurs, the kidney either retaining more or less accurately its natural form, or appearing like a mere appendix to the dilated pelvis and calices. Accumulations, of various kinds, in these cavities—pus and urine, and serous fluid—is the most common cause, in truth, of renal atrophy, and may diminish the quantity of actual kidney to such a degree that the stratum formed by it is not thicker, or very slightly thicker, than the external and internal membranes of the organ. Tumours of various kinds, developed in the substance of the kidney, determine its partial absorption; and growths or accumulations in surrounding parts—tumours of the liver or spleen, extensive abscesses in the lumbar cellular tissue, enlarged supra-renal capsules, &c.,—occasionally produce the same effect.

Atrophy may be confined to one of the renal substances, especially the cortical. It is sometimes connected with development of cysts, which in its turn may, according to M. Rayer, be or be not consecutive to partial inflammation of the renal tissue. The author has ascertained in the human subject and in animals the occasional connexion of certain partial atrophies with a very remarkable lesion of the tubular substance. The terminations of several mammillæ were in these cases flattened and infiltrated with a transparent substance of mucous or jelly-like appearance; the portions of cortical substance corresponding to the cases of these diseased mammillæ were depressed; and on the surface of the kidney little islets of cortical substance appeared resting on a grayish fibrous fundus.

Atrophy of a single kidney being almost always accompanied with supplementary development of the other organ produces no appreciable symptoms. When both organs are atrophous to a considerable extent there is derangement of the secretion of urine, and nervous phenomena, consisting of convulsions, trembling, and coma, are most commonly observed.

HYDRONEPHROSIS. M. Rayer proposes the name hydronephrosis for that condition of the kidney (referred to by others as *renal dropsy* and *hydrorenal distention*,) in which the urine accumulates slowly in the kidney without giving rise to inflammation of the pelvis or calices. The immediate cause of the accumulation is of course obstruction to the passage of the urine into the bladder; and the causes of this obstruction are, in turn: the presence of foreign bodies (calculi,) of entozoa (acephalocysts,) in the urinary passages; thickening and swelling of the walls of the ureters, or tumours protruding into their interior; vascular septa running across them; obliteration or contraction of those canals, &c. The distention in the majority of cases in the human subject affects both the pelvis and calices, and is commonly general. In the commencement when the dilatation is slight, the tissue of the kidney presents its natural degree of firmness; subsequently its surfaces grows tuberculated and misshapen, and the prominences on the surface feel soft and fluctuating. The pelvis and upper part of the ureter greatly distended usually form a pyriform tumour within the hilus of the kidney, having its point inferiorly.

When the organ is divided into an anterior and a posterior half the following appearances are seen. When the distension is slight, the pelvis is dilated, and the mammillæ more or less depressed; each calyx appears elongated and funnel-shaped in consequence of the widening of its base. The surface of the mammillæ becomes concave and rough to the touch; and in a more advanced stage the calices are enlarged into so many globular sacs, not communicating with each other, but opening all into the dilated pelvis. The surface of the pelvis is almost always of a pure white colour, and opaque; sometimes it presents lines having a mother-of-pearl lustre, and it scarcely ever contains visible blood-vessels. Atrophy of the renal substances occurs in the manner already described, and the cellulo-fibrous capsule of the kidney may undergo numerous changes. The liquid in the pelvis has always been found by M. Rayer to contain urea and a notable quantity of albumen when the hydronephrosis was of old standing; when the obstacle to the flow of urine was not complete, the fluid in the pelvis scarcely diffused from healthy urine. The absence of pus and other evidences of inflammation distinguishes these cases from pyelitis with distension.

Partial hydronephrosis, or the formation of small urinary cysts, may occur in one or more calices from the obstruction of their orifice of communication with the pelvis; or in the tubular cones from the obstruction of the opening of the mammillæ into the calices; or finally in the cortical substance. All these conditions M. Rayer observes are extremely rare in the human subject, but common in the ox.

When one kidney only is affected with hydronephrosis, the existence of the disease cannot be established until it has increased to such extent as to produce a soft fluctuating tumour in the renal region. This tumour may vary in size, according to M. Rayer, from that of the fist to that of the uterus in the last months of pregnancy. Its extent may generally be pretty accurately defined by percussion, and the tuberculated character of its surface ascertained by careful application of the hand. Tumours of this kind can only be mistaken in regard of their form for cysts of the kidney or accumulations of pus and blood in the pelvis and calices; in speaking of pyelitis we referred to the marks of distinction of the latter malady. Intercurrent inflammation may take place in a case of hydronephrosis and affect a more or less extensive surface of the pelvis; under these circumstances it would be very difficult to determine the true nature of the case, if then seen for the first time. M. Rayer has never met with an example of hydronephrosis terminating by the formation of a fistulous communication with the intestine, which is far from uncommon in true pyelitis.

In cases of hydronephrosis of one kidney, the disease appears, even where the distension is enormous, to be almost innocuous *in itself:* what constitutes the real danger is the chance of obstruction of the opposite ureter or inflammation of the opposite kidney. In a patient referred to by König in his chapter "Wassersucht der Nieren," a patient is stated to have lived twenty-three years with the disease; in another case observed by M. Rayer the first symptoms were noticed fifty years before death, which occurred at last from impaction of a calculus in the ureter of the opposite side. In cases of hydronephrosis of both organs the danger is proportional to the amount of atrophy of the renal tissues. All cases are more or less rapidly fatal, and it is well for the practitioner to be guarded in his prognosis, as the patient rarely keeps his bed until a few days before death, which occurs most commonly in a rapid and unexpected manner. A new-born infant affected with double hydronephrosis is not "*viable.*"

The only important point connected with the treatment of this malady is the question of the propriety of giving issue, by an opening in the renal region, to the accumulated fluid. König advised that these tumours should be punctured with the trocar, whenever they increased manifestly in size, and became fluctuating, just as in cases of ovarian dropsy. M. Rayer correctly criticises these directions. It is obvious that as hydronephrosis of one kidney is compatible with perfect exercise of the functions, the proceeding should not, under the supposition of the disease being simple, be put in force, more especially as the puncture has been known to induce fatal peritonitis. M. Rayer goes on to say, that the case is different if evidences of inflammation be perceptible in the tumour, with the possible tendency to softening and perforation of its walls; but the case, M. Rayer appears to forget, is then one of pyelitis, so that he would not admit the operation in a true case of hydronephrosis at all,—and in this he appears to have experience and reason on his side.

Cysts of the kidney. These are divided by M. Rayer into *simple*, *acephalocyst*, and *urinary*. Although containing matters of considerable interest, we shall not for the present undertake the analysis of this chapter.

Diseases of the blood-vessels. We pass to the diseases of the *renal arteries;* they appear to be neither numerous nor frequent. In the kidney of an old man M. Rayer found the renal artery of very large size, and presenting transverse deep furrows on its internal surface. The kidney fed by this artery was a sixth heavier than its fellow; however, as has already been hinted, no accurate ratio prevails between the size of the kidney and of its artery. The arteries are sometimes the seat of cartilaginiform and ossiform deposit; and M. Rayer has collected from different authors three very satisfactory cases of aneurism of these vessels.

The *emulgent veins* are the frequent subjects of congenital anomalies; it is unnecessary to refer particularly to their varieties. These vessels are not very commonly affected with inflammation, which exhibits itself in all its forms and degrees, from the formation of fibrinous coagula upwards. Renal phlebitis may coexist with inflammation, or some other affection of the kidneys; in other cases it is consecutive to inflammation of the vena cava, or ovarian vein. This connexion of the inflammation of the renal and ovarian veins was, we believe, first pointed out by that very indefatigable inquirer, Dr. Robert Lee.

The renal plexus, and the nerves distributed to the kidneys, may increase in volume in cases of hypertrophy of those organs; M. Rayer has figured an example of the fact. Whether they undergo diminution of size, in cases of atrophy, he is unable, from his own experience, to affirm. Nor can he throw any light on the interesting question, whether the nerves of the kidney may be the seat of neuralgia, independently of other affections, or of lesions of the neighbouring organs. No doubt the pain is not truly neuralgic in nephritic colic, but depends upon distension of the fibrous tissue of the ureter, more or less excoriated on its inner surface by the passage of a rough mass. Hysterical women sometimes complain of a pain in one or both renal regions, which has been duly described as neuralgia; but this M. Rayer is inclined to regard more as an affection of the spinal marrow, and of the lumbar nerves, than of the renal plexus. Aneurisms of the aorta sometimes occasion pain in the renal region, sufficiently intense to simulate an affection of the kidneys. The term nephralgia is, nevertheless, in spite of the want of precise knowledge of the subject, freely and boldly used by nosological writers. The whole subject stands in need of serious investigation.

HOMOLOGOUS FORMATIONS. M. Rayer next proceeds to the description of what he terms "homologous tissues," developed in, or in connexion with, the kidneys. The first of these considered is the cellulo-fibrous. The glands and ducts in the cortical substance are sometimes converted into a fibrous tissue of bluish white colour,—a condition, in some cases, the result of chronic inflammation, and in others of a lesion of the ducts of Bellini.

There are occasionally observable in the cortical substance, and more frequently in the tubular, certain whitish granules, of cartilaginous aspect and consistence; cartilaginous patches are sometimes found in the fibrous capsule.

The capsule may become ossified, present partial saline deposits, or assume altogether the appearance of a bony shell, resembling that sometimes formed by the pericardium round the heart. In rare cases the kidneys are the seat of "osteides;" that is, of masses composed fundamentally of fibrous tissue traversed by blood-vessels, and containing calcareous matter from place to place. These osteides are, according to M. Rayer, perfectly analogous to fibrous tumours of the uterus, and cannot be divided except with the saw. For some further descriptions of ossific deposit in the kidney we must refer our readers to the original volume.

The development of erectile tissue in the kidney is so rare, that M. Rayer has met with two examples only of it; in both instances the liver was affected with the same species of change. The erectile tissue appeared to consist of several small loculi, with veins ramifying between them [?]; no trace of scirrhus or encephaloid was to be discovered. Baillie and Lobstein appear also, from references made by M. Rayer, to have met with this kind of formation in the kidney.

FATTY KIDNEY. M. Rayer makes a very just division of the cases which have been described as examples of fatty degeneration of the kidney into two classes: one of these, characterized, according to this observer, by true fatty transformation of the organs, is very rare; the other, in which the morbid state consists of accumulation of fat round the organ which itself undergoes atrophy, is much more frequent. The kidney may besides become fatty in the same manner as the liver; Pascal has described a case in which the organ yielded a multitude of drops of oil under pressure. The following case, observed by M. Bricheteau, is interesting: A woman, aged forty-five, remarkably fat, was admitted under that physician at the Necker Hospital, with a series of symptoms, leading to the diagnosis of pneumonia. She had not passed any urine, according to her own account, for fifteen days; she was catheterised on three successive days, intervening between her admission and death, without a single drop of urine being found in the bladder. The kidneys appeared plunged in the midst of an immense quantity of fat; they retained their form and natural bulk, but were transformed into two masses of compact fat, in which some vestiges of tubular substance were traceable. The pelves, ureters, and bladder seemed healthy; the latter contained no urine.

Heterologous formations. The kidney is far from a very unfrequent depository of these; they occur here in every variety observed in other organs. In the description of them, M. Rayer gives evidence of his usual profound acquaintance with the subject of renal disease.

*Tuberculous disease.* Tubercles may occur in every one of the renal tissues; and sometimes, in consequence of acute or chronic inflammation of the surrounding substance they soften, the mass opens into the cavity of the renal pelvis, and the expulsion of the tuberculous matter may be followed by the formation of real cavities and fistulæ.

In the cortical substance the morbid matter is often deposited in small disseminated grains, of the size of a millet seed or less. These grains are commonly not permanent on the surface of the organ, nor adherent to its external membrane. They are distinguishable from the small pustular collections of pus occurring in some forms of nephritis by the absence of the narrow red circle which usually surrounds the latter. Tuberculous matter also occurs in the cortical substance in small compact masses of the size of a nut or an olive, smooth, of slightly yellow-white colour, and having altogether the appearance on section of a horse-chesnut. These masses are liable to soften; they are sometimes in immediate contact with the tissue of the kidney, in other instances separated from it by a very thin membrane, resembling the adventitious membrane found inclosing certain large abscesses of the liver.

As respects the tubular substance, the tuberculous matter may be deposited in it in grains, in masses, or as a sort of powder infiltrated in the substance of the mammillæ. These eminences may be destroyed by true tuberculous ulceration.

The condition of the renal tissue surrounding tuberculous matter varies. Though suffering pressure the tissue may have the appearance of perfect health, or it may be discoloured; when the kidneys contain a great abundance of tubercles, the intervening tissue appears injected, red with imbibed blood, and in cases of tuberculous nephritis, small collections of pus are found between the crude or softened tubercles.

In some cases of tuberculous deposition, the size of the kidneys appears manifestly increased; their bulk is rarely diminished. It has been said that both organs are generally attacked; of sixteen cases, however, observed by M. Rayer, six only were examples of double tuberculization; in seven of the ten remaining cases, the left organ suffered. When the kidneys are tuberculous, not only are the lungs almost invariably so, but also other parts of the genito-urinary system, the liver, the mesenteric glands, the intestines, &c.

The diagnosis of tuberculous disease of the kidneys is, it appears, even to M. Rayer, a matter of extreme difficulty. However the difficulty is by no means the same in all cases: it is infinitely greatest when the new product is seated exclusively in the proper substance of the kidneys themselves, and does not extend to the excretory canals. There is in truth, according to this observer, no distinctive condition of the urine in such cases: nor are the kidneys, except in rarest instances, sufficiently enlarged from their tuberculous infiltration of their substance to cause appreciable tumour externally. Pain in the seat of the organs may be produced by inflammation occurring around the tuberculous deposits; but the kidneys may become painful in tuberculous subjects without a deposition of the morbid matter within them.

When the tuberculous matter seated in the mammillæ undergoes softening, it is often detached and mingles with the urine; under these circumstances, or when the calices, pelvis, or ureters are infiltrated with this substance, the urine acquires certain characters which may announce the presence of tubercle in the urinary passages. At the moment of emission, it is more or less turbid, or at least it holds in suspension particles of organic non-fibrinous matter, which deposits with the salts of the fluid. Examined under the microscope, this sediment is found to be formed in great part of mucous globules and sometimes of blood-discs, together with an organic matter, which does not dissolve in dilute acids, as do the phosphates and urates. When this organic matter is closely scrutinised, it exhibits nothing but granules perfectly distinct from pus- and blood-globules. The quantity of this tuberculous matter, for such it is, varies extremely with different emissions, but never reaches the amount observed in the instance of purulent deposit. Another circumstance may occasionally occur to strengthen the diagnosis; the tuberculous matter sometimes accumulates in the upper part of the excretory passages, so as to cause obstruction to the escape of the urine, and so produce distension of the pelvis and calices with tumour appreciable by application of the hand and by percussion. Further, observation has frequently demonstrated the coexistence of tuberculization in the vertebræ and kidneys; the existence of tuberculous caries of the lower dorsal or upper lumbar vertebræ, *accompanied with changes in the constitution of the urine*, (provided neither paralysis nor retention of urine are present,) renders the existence of renal tubercles very probable.

The observations of M. Rayer have taught him that inflammation exercises no influence upon the development of tubercles in subjects who are not predisposed to this species of disease. Nay more, "the frequency of renal inflammation on the one hand, and the rarity of tubercles of the kidneys on the other, prove that it is doubtful, in the majority of cases at least, whether a local irritation [even in predisposed persons] has been the immediate cause of the tuberculous deposition."

It is useless to speak of the prognosis of tuberculous disease of the kidney, where it is a mere part of general tuberculization. When the disease affects the kidneys only, (a condition which must be, at the least, singularly rare,) the prognosis is less serious, and some advantage may, according to M. Rayer, be hoped from the use of an extremely strengthening diet, generous wine and preparations of iron, while the inflammatory symptoms are controlled by the frequent employment of gelatine baths.

CANCER OF THE KIDNEYS. This is a morbid state, which presents itself in different forms and degrees in these organs. Children appear to be rarely subjects of it; it is sometimes seen in adults; it is more common in advanced age. M. Rayer's own experience would lead him to consider the disease more common in males than females, an opposition to the general distribution of cancer in the two sexes, referrible no doubt to the causes of the greater frequency of all renal maladies in males.

Cancerous disease may be limited to the kidneys, or, as is much more common, it coexists with cancer in various other organs. In some instances it has appeared to M. Rayer that cancer of the kidney has originated under the influence of cancer of a neighbouring organ; thus he would explain the frequency of small cancerous masses in the right organ when the liver contains medullary sarcoma, and also "the well-established relations of cancer of the left kidney, with carcinomatous degeneration of the descending colon and fundus of the stomach."

*Encephaloid cancer* is the most common form of the disease in the kidney; fungus hæmatodes comes next in point of frequency, while it is yet more rare to detect true scirrhus of this organ. The disease almost always originates in the cortical substance, but it may extend to the tubular portion secondarily. The membranes of the kidney, the walls of the pelvis, and the blood coagulated in the renal veins may participate in the affection.

When the cancerous substance occurs in small masses few in number, the dimensions of the kidney are not manifestly increased; under other circumstances, the organ is capable of acquiring monstrous bulk, the surface then becomes irregularly tuberculated, with masses protruding to a considerable height in some instances.

M. Rayer has only found tubercle and cancer associated in the kidney in a very small number of cases; he has "more frequently seen tubercles in the lungs and encephaloid matter in the kidneys of the same individual." The author's experience upon this latter point appears to be at variance with the following statements of Dr. Walshe: "Tubercles and cancer rarely coexist. In fifty-two autopsies of cancerous subjects collected from various sources we found but three examples of the anatomical characters of phthisis. The difference of the ages at which the two diseases are most prevalent would lead us to expect a result of this kind, independently of any influence which the formation of one may have in excluding that of the other form of morbid matter."* We have recently heard that Professor Rokitansky, of Vienna, has expressed an opinion similar to that inculcated by Dr. Walshe. The point is an extremely interesting one, having obviously an important bearing with reference to the laws regulating diseased developments in general.

Cancer of the kidney may be almost completely, if not altogether, latent; that is, it may not have attained sufficient bulk to produce ex-

* Cyclopædia of Surgery, Art. Cancer, p. 623.

ternal tumour; it may not cause hematuria, and any pain to which it may give rise may be of a kind far from characteristic; indeed there is no species of renal pain essentially different from that caused by certain special diseases, by aneurism of the aorta, &c. The signification in which, we infer from this, M. Rayer uses the term latent, appears to us vague and indeed inaccurate; but we cannot stop to argue the point. After a certain period has elapsed, the kidney enlarges sufficiently to form an appreciable tumour, variable in size and commonly presenting, as we have seen, irregular protuberances on its surface. Hematuria also sets in and recurs irregularly, being occasionally interrupted, among other causes, by the obstruction of coagula in the ureter. The urine is turbid; the colour deep; the odour fetid. Retraction of the testicle, which so frequently occurs in cases of pyelitis with obstruction of the urine, is rarely met with in cancer.

The general symptoms of the disease are those characteristic of carcinoma generally. Of its causes nothing appears to be distinctly known; the various maladies of the urinary organs, and especially their inflammations, have no manifest influence on its production.

The occurrence of hematuria in a subject who has laboured under pain in the renal region, and who has not passed gravel nor suffered from complete or incomplete retention of urine, is of some importance in respect of the diagnosis of the disease. Without hematuria to guide us, it is indeed often impossible to ascertain the real seat of cancerous disease in the kidney, when sufficiently extensive to produce considerable tumour.

True *melanosis* of the kidney has only been observed by M. Rayer in cases of the melanic diathesis, when formations of the same kind existed in considerable number in the skin or in the subcutaneous cellular tissue, the lungs, or other organs; and even under these circumstances there were but slight traces of melanosis in the kidney. Symptomatically considered the disease is unimportant, and M. Rayer furnishes no information of novel character respecting the anatomical peculiarities of the disease.

ENTOZOA IN THE KIDNEYS. The chapter on the entozoa found in the kidneys commences by some very just observations upon the general inaccuracy and unsatisfactory character of the statements of older authors on the subject of worms voided through the urinary passages. The entozoa, which are indubitably forced in the kidneys in certain cases, are the acephalocyst, the strongylus gigas, the spiroptera hominis, and the dactylius aculeatus.

The strongylus is distinguished by its cylindrical elastic body attenuated at both extremities, generally several inches long in the human subject, and of a blood-red colour when alive; this colour is discharged by keeping the animal in water or alcohol. These worms bear some resemblance in form and size to lumbrici, and have, it would appear, been mistaken for them. Of the rarity of the strongylus, a sufficient proof (in addition to common experience) is furnished by the fact that M. Rayer has examined more than 3000 human kidneys and more than 500 kidneys in the dog without finding a single one. The symptoms determined by the presence of strongyli in the kidneys are extremely like those produced by renal calculi, namely, pain in the kidneys, frequently hematuria, occasionally retention of urine in the pelvis and calices, and as a result of this the development of a tumour in the lumbar region. Tumours of this kind may burst spontaneously, externally, or be opened (as has been done) by the surgeon. During life the existence of strongyli in the kidneys cannot be demonstrated unless by the expulsion of one or more of these worms; for as we have first seen, the symptoms they determine are simply those of pyelitis with retention. Nothing is known respecting remedies capable of determining the expulsion of strongyli during life; their rare occurrence sufficiently explains this deficiency of knowledge.

The spiroptera is an entozoon found occasionally between the coats of the stomach in mammalia, and especially in birds; there appears to be but one case on record in which individuals of this species were voided with the urine. We allude to the case reported by Mr. Lawrence in the second volume of the Medico-Chirurgical Transactions; Bremser and Rudolphi having both expressed their opinion that the worms voided in this case were spiropteræ; M. Rayer transcribes the narrative, but has no additional information to afford. His account of the dactylius aculeatus is copied from Mr. Curling's paper on the subject.

Malformations of the kidneys. We proceed to notice briefly M. Rayer's observations on these. Numerous are the cases of three kidneys in the human subject described by authors; nor are examples of the existence of four of these organs wanting. The only point of physiological interest connected with this superabundance of kidney is that no evidence exists of its ever being attended with unnaturally great secretion of urine.

The total absence of kidneys has been several times noticed in the fœtus, occasionally in the infant at birth, and one case is recorded of such absence in a young girl. This last almost incredible history is related by M. Moulon, physician to the Trieste Hospital, of a person aged fourteen, who died of chronic enteritis; among other malformations, there were neither ureters nor kidneys to be found; but the caliber of the umbilical vein greatly exceeded that usual in adults. The girl had from her birth been subject to an inconvenience of very troublesome character; there flowed continually from the umbilicus a fluid closely resembling urine, and emitting so strong a smell that it was impossible to change the linen covering the part sufficiently often. The observer of the case supposes that the anatomical conditions now noticed (the bladder was also wanting) justify the idea that the blood freed itself in the liver from the principles entering into the composition of the urine, and that these were conveyed by the umbilical vein to the umbilicus, whence they were finally excreted. If, observes M. Rayer, the case related by M. Moulon were accurately reported, it would render less incomprehensible than they now are certain cases of anuria on record, one for example described by M. Vieuisseux, of Geneva, in which total suppression of urine existed for seventeen months, at the close of which period the discharge returned, without the patient having suffered from any alarming symptoms. But M. Rayer is inclined to believe that M. Moulon's patient was really affected with extrophia vesicæ, and that the kidneys, "peut-être déplacés," were not looked for with sufficient care. This "peut-être déplacés" is really too bad; as if it is possible to conceive that a man holding the position of physician to a large hospital in an important city could be so flagrantly ignorant, especially when all his senses must have been alive to the extraordinary nature of the case, as not to know the kidneys, if they had been in their natural place. Really the *chances*, to say the least, are against M. Rayer.

Numerous authentic cases are to be found of absence of one kidney. The remaining organ has generally been found larger than natural, and sometimes of double its natural weight. It is either found in its natural place, or a little higher or lower; where a single kidney has been described as seated crosswise on the spine, there, no doubt, existed two kidneys fused, as it were, into one. There is a very curious example of "like master, like man," given by M. Rayer under this head: Cabrole opened the body of a professor at Montpellier and found but one kidney; one of the servants of this gentleman was subsequently found likewise to possess but a single kidney, or at least apparently but one, his case being, according to a conjecture of M. Rayer, probably an example of fusion of the two organs into one.

The kidneys are the occasional subjects of anomalies of situation, either permanent or non-permanent. When they are fixed permanently in an abnormal situation, this state may have been congenital or accidental; the subject is generally tolerably well understood, and we shall not dwell upon it here. Non-permanent displacement or mobility of the kidney is a state less known, and possessing much more practical pathological interest; we shall, therefore, give our readers the benefit of M. Rayer's observations upon it. Mobility of the kidneys is that condition which allows of their changing their position downwards or forwards, or of their being pushed upwards under the liver or backwards, and is the cause of various symptoms; for example, habitual pain in the abdomen and corresponding lower extremity, pain which has been mistaken for nervous colic, hypochondrical pain or lumbar sciatic neuralgia. This condition of the kidney is far more frequent than is commonly imagined; is met with almost exclusively on the right side, and occurs more frequently in females than males. In the majority of cases there is coexistent with it enlargement of the liver, with displacement of the intestine or uterus; it may also be a result of a particular arrangement of the peritoneum, of a deviation of the kidney, or of a flexuous state of its vessels, &c. Frequent pregnancy, laborious efforts to raise heavy loads, &c. have in some cases appeared to be its immediate cause, in other instances no direct cause has been discovered.

The most common symptoms of this state of the kidney are pain in the right lumbar region, sometimes extending in the direction of the lumbar and crural nerves, with an habitual sensation of weakness and discomfort in the abdomen. The pain sometimes ceases, and may then be excited by pressing the kidney with the fingers of the right hand, while the left supports the loins behind. Peritonitis sometimes complicates the case, probably induced by the traction exercised on the peritoneum by the displaced organ. Patients sometimes discover the tumour themselves, and naturally feel much apprehension as to its character. Of two medical men thus affected, and in whom M. Rayer was the first to detect the true nature of the case, one had left the profession altogether from the dread of having serious organic disease.

Experience has proved to M. Rayer that in general all that needs to be done in these cases is to support the abdomen with a belt, which either removes the pain altogether or at least renders it very bearable. In two cases where this renal displacement coexisted with procidentia of the uterus, great benefit was derived from constantly observing the recumbent posture and the use of sulphurous *douches*. When the pain is very acute, and there are signs of either peritonitis or enteritis, local bleeding and emollient applications should be employed. All violent exercises, dancing, riding, leaping, &c. are of course to be avoided; and constipation carefully guarded against.

With this brief notice of a very curious and practically interesting condition of the kidney, (of which we had the opportunity, during a recent visit to Paris, of observing an example, in M. Rayer's wards,) we for the present take leave of this laborious and estimable writer, with the hope that the appearance of his fourth and concluding volume will soon enable us to introduce him once again to the notice of our readers.

---

